# Presynaptic [Ca^2+^] and GCAPs: aspects on the structure and function of photoreceptor ribbon synapses

**DOI:** 10.3389/fnmol.2014.00003

**Published:** 2014-02-06

**Authors:** Frank Schmitz

**Affiliations:** Department of Neuroanatomy, Institute for Anatomy and Cell Biology, Medical School Homburg/Saar, Saarland UniversitySaarland, Germany

**Keywords:** photoreceptor, ribbon synapse, synaptic ribbon, RIBEYE, Ca^2+^, GCAP2

## Abstract

Changes in intracellular calcium ions [Ca^2+^] play important roles in photoreceptor signaling. Consequently, intracellular [Ca^2+^] levels need to be tightly controlled. In the light-sensitive outer segments (OS) of photoreceptors, Ca^2+^ regulates the activity of retinal guanylate cyclases thus playing a central role in phototransduction and light-adaptation by restoring light-induced decreases in cGMP. In the synaptic terminals, changes of intracellular Ca^2+^ trigger various aspects of neurotransmission. Photoreceptors employ tonically active ribbon synapses that encode light-induced, graded changes of membrane potential into modulation of continuous synaptic vesicle exocytosis. The active zones of ribbon synapses contain large electron-dense structures, synaptic ribbons, that are associated with large numbers of synaptic vesicles. Synaptic coding at ribbon synapses differs from synaptic coding at conventional (phasic) synapses. Recent studies revealed new insights how synaptic ribbons are involved in this process. This review focuses on the regulation of [Ca^2+^] in presynaptic photoreceptor terminals and on the function of a particular Ca^2+^-regulated protein, the neuronal calcium sensor protein GCAP2 (guanylate cyclase-activating protein-2) in the photoreceptor ribbon synapse. GCAP2, an EF-hand-containing protein plays multiple roles in the OS and in the photoreceptor synapse. In the OS, GCAP2 works as a Ca^2+^-sensor within a Ca^2+^-regulated feedback loop that adjusts cGMP levels. In the photoreceptor synapse, GCAP2 binds to RIBEYE, a component of synaptic ribbons, and mediates Ca^2+^-dependent plasticity at that site. Possible mechanisms are discussed.

## INTRODUCTION

### PRESYNAPTIC [Ca^2+^]: ASPECTS ON THE STRUCTURE AND FUNCTION OF PHOTORECEPTOR RIBBON SYNAPSES

Changes in presynaptic free cytosolic Ca^2+^ ions trigger important functions in the presynaptic terminals. These include synaptic vesicles exocytosis and the mediation of various forms of synaptic plasticity (Bliss and Collingridge, 1993; Malenka and Nicoll, 1999; Neher and Sakaba, 2008; Kawamoto et al., 2012; Rizo and Südhof, 2012; Catterall et al., 2013; Südhof, 2013). Ca^2+^ ions also have fundamental functions in photoreceptor synapses. Photoreceptors employ tonically active ribbon synapses that encode light-induced, graded changes of membrane potential into modulation of continuous synaptic vesicle exocytosis to transmit the light-induced signals to the inner retina. The active zones of ribbon synapses contain large electron-dense structures, synaptic ribbons, that are associated with large numbers of synaptic vesicles. Synaptic ribbons have a key importance for signaling at the ribbon synapse. This review focuses on the regulation of [Ca^2+^] in presynaptic photoreceptor terminals and on the function of a particular Ca^2+^-regulated protein, the neuronal calcium sensor (NCS) protein GCAP2 (guanylate cyclase-activating protein-2). GCAP2, an EF-hand-containing, Ca^2+^-binding protein is strongly expressed in photoreceptors and plays multiple roles in the outer segments (OS) and in the photoreceptor synapse.

### PHOTORECEPTORS

**FIGURE 1:**
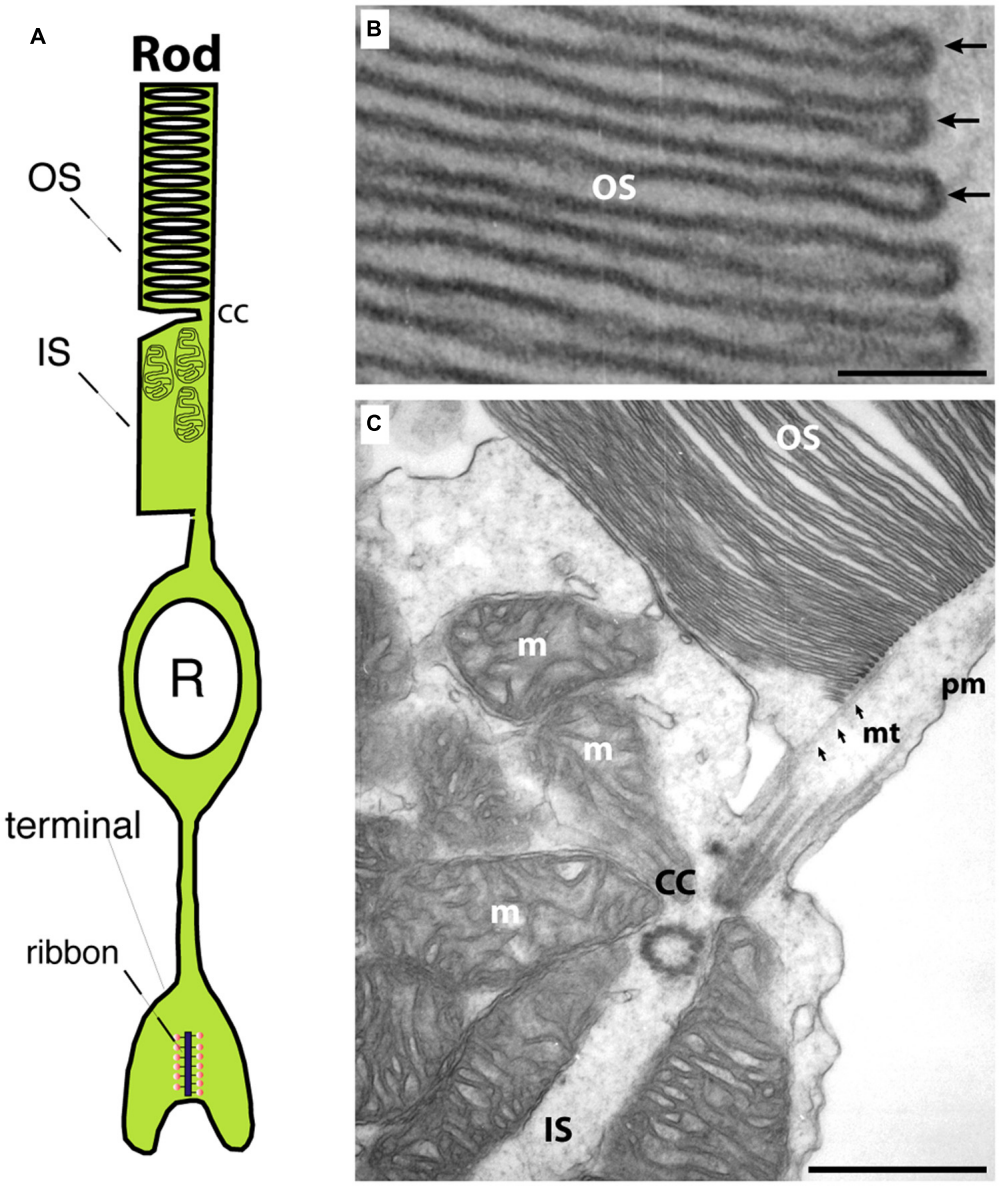
**(A)** Photoreceptors show a bipolar morphology. A distal process forms the outer segment (OS) and the inner segment (IS). The OS is the place where phototransduction takes place. The IS represents the metabolic center of the photoreceptor. An inner, vitread process ends in the presynaptic terminal that contains a synaptic ribbon at the active zone. **(B)** Transmission electron micrograph of an outer segment (OS). Densely packed arrays of membrane disks can be observed. The arrows in **(B)** point to individual disks. In the OS, phototransduction occurs and GCAP proteins perform a Ca^2+^-dependent feedback on guanylate cyclases. **(C)** Transmission electron micrograph at the junctional zone between OS and IS. OS and IS are connected by the connecting cilum (cc) that organizes mt-dependent vesicle transport into the outer segment (Sung and Chuang, 2010). OS, outer segment; IS, inner segment; m, mitochondrion; cc, connecting cilium linking OS and IS; mt, microtubule; pm, plasma membrane. Scale bars: 50 nm **(B)**, 250 nm **(C)**.

**FIGURE 2:**
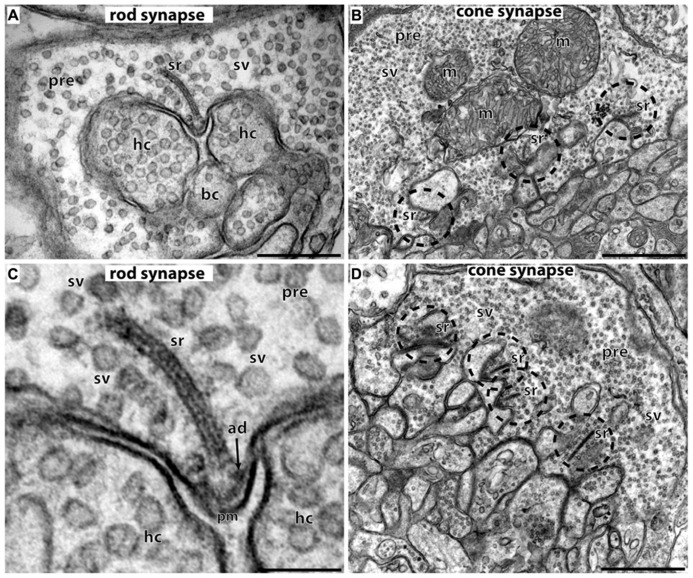
**Photoreceptor ribbon synapses.** In **(A,C)** rod photoreceptor ribbon synapses are shown by transmission electron microscopy; in **(B,D)** cone photoreceptor ribbon synapses. **(A,B)** The large presynaptic terminals are filled with numerous synaptic vesicles (sv). The active zone is characterized by specialized presynaptic densities, the arciform densities (ad). The synaptic ribbon (sr) is anchored to the arciform density. The synaptic ribbon is associated with large numbers of synaptic vesicles. Opposite to the active zones are the dendritic tips of horizontal cells (hc) and bipolar cells (bc) that contain ionotropic and metabotropic glutamate receptors for signaling. The dendritic tips are located within an invagination of the presynaptic terminal. Cone synapses **(B,D)** are larger in diameter than rod synapses and contain multiple active zones (dashed circles) and multiple synaptic ribbons (sr). sr, synaptic ribbon; sv, synaptic vesicles; ad, arciform density; pre, presynaptic terminal; m, mitochondrium; hc, dendritic tips of horizontal cells; bc, dendritic tips of bipolar cells; pm, presynaptic plasma membrane. Scale bars: 400 nm **(A)**, 1 μm **(B)**, 150 nm **(C)**, 800 nm **(D)**.

Photoreceptors are the principal light-sensitive neurons in the outer retina. They detect incoming light quanta (photons) and communicate the information to the inner retina for further processing. Rod photoreceptors operate at low light intensities and are capable of single-photon-detection. Cone photoreceptors operate at daylight conditions and mediate color vision (Abramov and Gordon, 1994). Both types of photoreceptors display a bipolar morphology with two processes emanating from opposite ends of the photoreceptor cell body (**Figure 1A**). (1) An outer process forms the outer segment (OS) that is responsible for phototransduction. During this process, light-sensitive photopigments in the OS transform light quanta into a change of the photoreceptor membrane potential. (2) An inner cell process emerges from the vitread portion of the photoreceptor soma. This inner process ends in a specialized presynaptic terminal that contains synaptic ribbons in their active zones (**Figures 1A** and **2**). At these ribbon-type active zones, signals generated by the absorption of light quanta in the OS are continuously transmitted to the inner retina (for review, see Wässle, 2004; Nassi and Callaway, 2009; Lee et al., 2010).

### Ca^2+^-DEPENDENT REGULATION OF PHOTOTRANSDUCTION IN PHOTORECEPTOR OUTER SEGMENTS

The OS contain dense and regular intracellular staples of membrane disks (for rods, **Figure 1**; for review, see Mustafi et al., 2009). These membrane-specializations are enriched with light-sensitive opsin-containing photopigments (Burns and Baylor, 2001; Chen, 2005). As light quanta are detected by the photopigments in the OS, a signal transduction cascade is initiated that finally leads via G-protein-mediated activation of a phosphodiesterase, phosphodiesterase 6 (PDE6), to the hydrolysis of cGMP (Burns and Baylor, 2001; Chen, 2005; Dell’Orco and Koch, 2010). The drop in cGMP leads to the closure of cyclic nucleotide-gated cation channels (CNG-channels) in the plasma membrane, a non-selective cation channel through which both Na^+^- and Ca^2+^-ions can pass from extra- to intracellular space along their electrochemical gradient. The light-induced closure of the CNG-channels generates a hyperpolarization response. The membrane potential ranges from ≈-40 mV in the dark to ≈-70 mV in bright light. The phototransduction system has a high dynamic range that is adjusted – not exclusively, but to a considerable extent – by Ca^2+^-dependent processes (Arshavsky, 2002; Korenbrot and Rebrik, 2002; Sharma, 2002, 2010; Koch, 2006; Artemyev, 2008; Stephen et al., 2008; Dell’Orco and Koch, 2010; Sharma and Duda, 2012). The light-induced drop of Ca^2+^ is sensed by Ca^2+^-binding, EF-hand-containing proteins, the guanylate cyclase-activating proteins (GCAPs; Korenbrot and Rebrik, 2002; Palczewski et al., 2004; Koch, 2006; Sakurai et al., 2011; Sharma and Duda, 2012). GCAPs are members of the family of NCS proteins (Sharma, 2002, 2010; Koch, 2006; Burgoyne, 2007; Baehr and Palczewski, 2009; Koch et al., 2010; Schmitz et al., 2012). GCAP2 (as well as its close relatives GCAP1 and GCAP3; for review, see Palczewski et al., 2004) contains four EF-hands. The first EF-hand is not able to bind Ca^2+^ due to exchange of critical amino acids (Palczewski et al., 2004; Koch, 2006; Burgoyne, 2007; Schmitz et al., 2012). Thus, under light stimulation, when Ca^2+^ is low, GCAPs activate the photoreceptor retinal guanylate cyclase (ret-GC) thus restoring cGMP baseline levels (Burns and Baylor, 2001; Sharma, 2002, 2010; Chen, 2005; Sharma and Duda, 2012). In the dark, at elevated Ca^2+^, GCAPs inhibit ret-GCs activity and prevent over-production of cGMP. These Ca^2+^-/GCAP-dependent processes set the dynamic range of phototransduction and keep the OS and phototransduction operational at different levels of photon fluxes (Koch, 2006; Stephen et al., 2008; Sharma and Duda, 2012). GCAPs are myristoylated at their N-terminus. Remarkably, in contrast to other members of the NCS family, GCAPs do not perform a Ca^2+^-dependent myristoyl switch at the N-terminus (Stephen et al., 2007). Instead, the N-terminal myristoyl-group remains buried in the inside of the molecule both in the Ca^2+^-bound and Ca^2+^-free state where it is essential to stabilize the protein (Stephen et al., 2007). Unusually, the C-terminus of GCAP2 performs a Ca^2+^-dependent conformational change (Peshenko et al., 2004).

### THE PHOTORECEPTOR RIBBON SYNAPSE OF THE MATURE RETINA: ULTRASTRUCTURE AND MOLECULAR ANATOMY OF THE SYNAPTIC RIBBON COMPLEX

Photoreceptors are non-spiking neurons and communicate light-induced, graded changes of membrane potential into modulations of tonic neurotransmitter release rates at the presynaptic terminals (Heidelberger et al., 2005; Matthews and Fuchs, 2010; Mercer and Thoreson, 2011). For this purpose, photoreceptor terminals are equipped with large active zones to which conspicuous presynaptic specializations, the synaptic ribbons, are attached (for review, see tom Dieck and Brandstätter, 2006; Schmitz, 2009; Matthews and Fuchs, 2010; Mercer and Thoreson, 2011; Schmitz et al., 2012).

**FIGURE 3:**
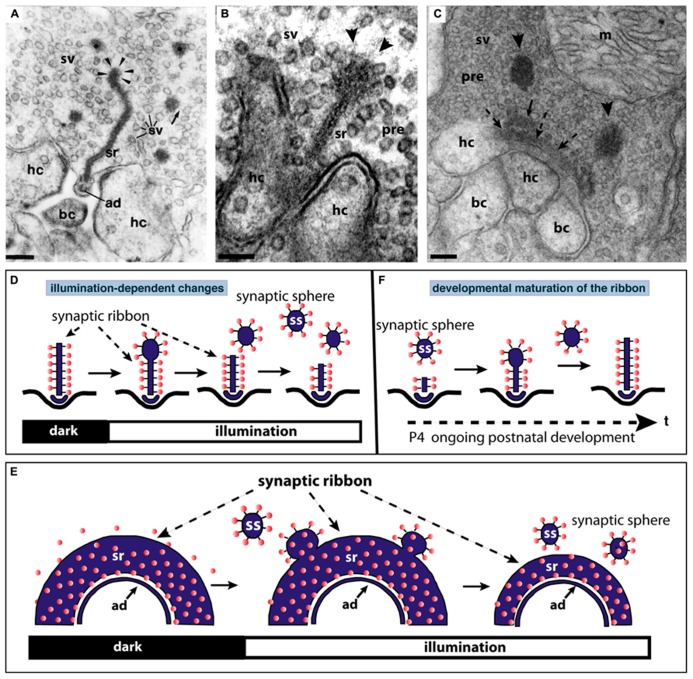
**The synaptic ribbon is a dynamic structure, both in the mature retina (A,B,D,E) as well as in the immature retina during early postnatal retinal development (C,F).** Panels **(A,B)** show disassemblying synaptic ribbons in the mature retina. The mature, bar-shaped synaptic ribbon disassembles via spherical structures, the synaptic spheres (arrowheads in **A,B**). Disassembly into synaptic spheres occurs at the cytosolic, non-plasma membrane-anchored end of the synaptic ribbon (arrowheads in **A,B**). At this end, the synaptic ribbon appears enlarged before the spherical synaptic spheres detach from it **(A,B)**. The spherical synaptic ribbons are still associated with synaptic vesicles via thin connections. But they are no longer anchored to the presynaptic plasma membrane. Panel **(A)** is modified from Schmitz and Drenckhahn (1993) with permission from Springer-Verlag, Berlin, Heidelberg, Germany. **(C)** Assembly of the mature, bar-shaped synaptic ribbon during postnatal development. The arciform density (ad) is clearly visible at the active zone (dashed arrowheads in **C**). In the synapse shown in **(C)**, the assembly of the synaptic ribbon just started. A small piece of the ribbon is already assembled (small black arrow in **C**) and anchored at the arciform density. Most of the ribbon is still present as immature synaptic spheres (arrowheads) located in vicinity to the small anchored ribbon primer (arrow in **C**). Later in development, the spherical synaptic ribbons (arrowheads) coalesce with the anchored ribbon primer (small black arrow in **C**) to form the mature, bar-shaped synaptic ribbon. RIBEYE–RIBEYE interactions might mediate this process. Panel **(C)** is taken from Schmitz et al. (2006) with permission from PNAS (copyright (2006) National Academy of Sciences, USA). Panels **(D,E)** schematically depict the illumination-induced disassembly of the synaptic ribbon (see also **A,B**). Panel **(F)** summarizes the possible sequence of synaptic ribbon assembly that occurs during postnatal development. sr, synaptic ribbon; ss, synaptic sphere; pre, presynaptic, sv, synaptic vesicle; bc, bipolar cell dendritic tip; hc, horizontal cell dendritic tip; m, mitochondrium; P4, postnatal day 4. Arrowheads in **(A,B)** point to synaptic spheres forming at the distal, non-membrane-anchored end of the synaptic ribbon. Dashed arrows in **(C)** point to the arciform density, which is the presynaptic density in ribbon synapses. The spheres colored in red in **(D–F)** represent synaptic vesicles. Panels **(D–F)** were re-drawn based on figures by Adly et al. (1999) and Spiwoks-Becker et al. (2004). Scale bars: 100 nm **(A–C)**.

The synaptic ribbon is a large, electron-dense structure that is associated with large numbers of synaptic vesicles along its entire surface (**Figure 2**; see also Schmitz, 2009). In photoreceptor ribbon synapses, the synaptic ribbon usually appears as a bar-shaped structure of several 100 nm in length in the transmission electron microscope (Schmitz, 2009; Mercer and Thoreson, 2011; Schmitz et al., 2012). Three-dimensional reconstructions of the synaptic ribbon revealed that this bar-shaped profile is a cross-section of a plate-shaped structure of the synaptic ribbon (**Figures 2** and **3E**; Rao-Mirotznik et al., 1995; for review, see also Schmitz, 2009; Mercer and Thoreson, 2011; Schmitz et al., 2012). Furthermore, the synaptic ribbon is bended in a horseshoe-shaped manner along the invaginated presynaptic plasma membrane of the photoreceptor terminal. The horseshoe-shaped synaptic ribbon can have a depth of about 1 μm (Schmitz, 2009; Schmitz et al., 2012). Rod photoreceptor presynaptic terminals usually possess a single active zone and a single synaptic ribbon; cone presynaptic terminals possess multiple active zones and multiple, though typically smaller synaptic ribbons (**Figure 2**; Wässle, 2004; Schmitz, 2009; Mercer and Thoreson, 2011; Regus-Leidig and Brandstätter, 2012; Schmitz et al., 2012). In photoreceptor ribbon synapses, synaptic ribbons are anchored to the arciform density. The arciform density corresponds to the presynaptic projections of conventional synapses (tom Dieck and Brandstätter, 2006; Schmitz, 2009; Mercer and Thoreson, 2011; **Figure 2**). At the plasma membrane of the active zone, L-type voltage-gated Ca^2+^-channels (VGCC) are clustered. Ca^2+^-influx through these channels triggers release at the photoreceptor synapse (see below). The active zone with the attached synaptic ribbon is opposed by the tips of multiple postsynaptic dendritic endings from horizontal and bipolar cells. These dendritic tips are located in a cavity formed by an invagination of the presynaptic photoreceptor terminal. The dendritic tips contain metabotropic glutamate receptors (in case of invaginating ON bipolar cells) or ionotropic glutamate receptors (in case of horizontal cells and OFF bipolar cells; Nomura et al., 1994; De Vries and Schwartz, 1999; De Vries, 2000; Wässle, 2004; De Vries et al., 2006; for review, see Dhingra and Vardi, 2012). Retinal bipolar cells, photoreceptor-like neurons in the pineal gland, inner ear hair cells, and vestibular hair cells also build ribbon synapses in mammals (Moser et al., 2006; Schmitz, 2009; Matthews and Fuchs, 2010; Mercer and Thoreson, 2011; Safieddine et al., 2012; Schmitz et al., 2012).

RIBEYE is a unique and major component of synaptic ribbons (Schmitz et al., 2000, 2012; Wan et al., 2005; Schmitz, 2009; Mercer and Thoreson, 2011). RIBEYE is the only known protein specific to synaptic ribbons (Schmitz et al., 2000; Magupalli et al., 2008; for review, see Schmitz, 2009; Mercer and Thoreson, 2011; Schmitz et al., 2012). RIBEYE consists of a unique A-domain that is specific to synaptic ribbons and a B-domain that is largely identical to CtBP2. Using different promoters, RIBEYE and CtBP2 are transcribed from the same gene (Schmitz, 2009). While CtBP2 is expressed in most if not all cells; the expression of RIBEYE expression is limited to cells that form ribbon synapses. Particularly the A-domain contains many interaction sites, at which RIBEYE can bind to neighboring RIBEYE molecules. These multiple RIBEYE–RIBEYE interactions could allow the assembly of multiple RIBEYE molecules into ribbon-like structures (Magupalli et al., 2008; for review, see Schmitz, 2009; Mercer and Thoreson, 2011; Schmitz et al., 2012). These primitive, spherical ribbon precursors could then assemble into mature, bar-/plate-shaped synaptic ribbons in mature photoreceptors (see below). RIBEYE appears to be the main building block of synaptic ribbons. Additional proteins are needed to generate mature, functional synaptic ribbons (see below).

Both RIBEYE(B)-domain and CtBP1/CtBP2 bind NAD(H) (Schmitz et al., 2000). In case of CtBP1/CtBP2, binding of NADH regulates interaction with nuclear proteins (Fjeld et al., 2003). This NAD(H)-regulated interaction can switch on or off distinct target genes relevant, e.g., for cellular migration, adhesion, and apoptosis (Paliwal et al., 2006, 2007, 2012; Chinnadurai, 2009). In the synapse, NAD(H) promotes interaction of RIBEYE with other synaptic proteins, e.g., the NCS protein GCAP2 (Venkatesan et al., 2010; López-del Hoyo et al., 2012). RIBEYE–GCAP2 interaction appears to be relevant for mediating Ca^2+^-dependent effects on synaptic ribbon dynamics (see below). Other protein–protein interactions of RIBEYE do not depend on NADH, e.g., RIBEYE–Munc119 interaction (Alpadi et al., 2008) or have not been analyzed for their NADH-dependence, e.g., in case of RIBEYE-Bassoon (tom Dieck et al., 2005). While RIBEYE appears to be the only unique and major protein of the synaptic ribbon, there are other ribbon proteins which are also present in the active zone of non-ribbon containing synapses (for review, see tom Dieck and Brandstätter, 2006; Schmitz, 2009; Mercer and Thoreson, 2011; Schmitz et al., 2012). Bassoon is localized in the active zone of photoreceptors, bipolar cells, and hair cells (in addition to the active zone localization of conventional synapses) and is considered as an important anchor for the synaptic ribbon in ribbon synapses. Bassoon knockout mice display non-plasma membrane-anchored “floating” ribbons in photoreceptor and hair cells (Khimich et al., 2005; tom Dieck et al., 2005), though not in retinal bipolar cells. Bassoon is also present in retinal bipolar cells (Deguchi-Tawarada et al., 2006). Therefore, additional anchoring mechanisms for the synaptic ribbon might exist. Bassoon was also been reported to be linked to presynaptic Cav-channels recently (Nishimune et al., 2004, 2012; see also below). Piccolo, like bassoon a very large multidomain-protein, is present both in conventional and ribbon-type synapses. Recently, a splice variant of piccolo was described, named piccolino, that is preferentially expressed at ribbon synapses (Regus-Leidig et al., 2013). Proteins known to be essential for synapse function in conventional synapses are the multi-domain rab3-interacting molecule (RIM) protein family (Wang et al., 1997; Kaeser et al., 2011, 2012; for review, see Südhof, 2013). These proteins have been localized to the synaptic ribbon. The long isoforms of RIM contain a N-terminal Zn-finger, central PDZ-domain and two C2-domains (Südhof, 2012a,b, 2013) that enable them to perform key synaptic functions. RIM proteins control synaptic vesicle docking and vesicle priming as well as Ca^2+^-channel localization (for review, see Südhof, 2012b, 2013). Their function in the ribbon synapse is less clear. RIM2 appears to be localized at the base of the synaptic ribbon and RIM1 at the body of the synaptic ribbon (tom Dieck et al., 2005). RIM proteins interact with Munc13 proteins in conventional synapses to mediate vesicle priming (Deng et al., 2011). While in conventional synapses, RIMs interact with Munc13-1; Munc13-1 is missing in photoreceptor synapses (Schmitz et al., 2001; Cooper et al., 2012). Instead of Munc13-1, ubMunc13-2 is expressed in photoreceptor synapses (Cooper et al., 2012). Other active zone proteins [e.g., CAST/ELKS; RIM-binding proteins (RIM-BPs)] are also present at photoreceptor ribbon synapses partly differentially distributed in distinct sub-compartments of the synapse (for review, see tom Dieck and Brandstätter, 2006; Mercer and Thoreson, 2011; Schmitz et al., 2012).

### SYNAPTIC RIBBONS: FUNCTIONAL CONSIDERATIONS

The synaptic ribbon is the most prominent structural feature of photoreceptor ribbon synapses. The function of the synaptic ribbon is still unclear. The synaptic vesicles attached to the synaptic ribbon have been functionally correlated to different synaptic vesicle pools (for review, see Heidelberger et al., 2005; Matthews and Fuchs, 2010; Mercer and Thoreson, 2011). Total internal reflection fluorescence (TIRF) microscopy studies of individual synaptic vesicles demonstrated that the base of the ribbon is a hotspot of synaptic vesicle exocytosis (Zenisek et al., 2000; Chen et al., 2013). The basal row of synaptic vesicles attached to the synaptic ribbon represents the vesicle pool that can be immediately released (the “RRP,” readily releasable pool). Therefore, one role of the ribbon might be to position this basal row of synaptic vesicles in a “strike-distance” for SNARE proteins to rapidly execute Ca^2+^-triggered exocytosis (Zenisek et al., 2000). The synaptic ribbon seems to be needed for both fast, synchronous release as well as for slow, asynchronous release components (Khimich et al., 2005; Zenisek, 2008; Frank et al., 2010 Snellman et al., 2011).

Exocytosis was frequently observed at ribbon-containing sites if exocytosis was induced by mild stimuli, e.g., weak depolarizations (Chen et al., 2013). If longer depolarizations were applied, exocytosis also occurred at non-ribbon-containing sites, i.e., in some distance from the presynaptic Ca^2+^-channels (Midorikawa et al., 2007; Zenisek, 2008; Chen et al., 2013). Exocytosis of synaptic vesicles at these non-ribbon-containing sites could contribute to the asynchronous release component (Midorikawa et al., 2007; Zenisek, 2008).

The large dimensions of the synaptic ribbon allow many vesicles to be docked at the base of the synaptic ribbon (Matthews and Fuchs, 2010; Mercer and Thoreson, 2011; Schmitz et al., 2012). This is quite likely necessary to support coordinated multivesicular vesicle fusion at the synaptic ribbon (Glowatzki and Fuchs, 2002; Singer et al., 2004; Khimich et al., 2005; Graydon et al., 2011). The synaptic vesicles attached to the synaptic ribbon in a more distant position (also denoted as “tethered vesicles”; Heidelberger et al., 2005; Matthews and Fuchs, 2010; Mercer and Thoreson, 2011) could contribute to the synaptic vesicle pool that is released with a slower kinetics, the slowly releasable pool (“SRP”). The tethered synaptic vesicles could possibly move down toward the base of the synaptic ribbon before they finally fuse at the active zone (“conveyor belt” hypothesis of the synaptic ribbon; Lenzi and von Gersdorff, 2001; Parsons and Sterling, 2003).

Recently, it was shown that the synaptic ribbon has also an important role in the replenishment of synaptic vesicles (Jackman et al., 2009; Snellman et al., 2011; Chen et al., 2013; see also below). Regulation of synaptic vesicle replenishment could be a major function of synaptic ribbons (Jackman et al., 2009; Chen et al., 2013). It was shown that tonic release at the photoreceptor ribbon synapse leads to vesicle depletion at the base of the ribbon. Based on that suggestion, the ribbon would work as a capacitor for synaptic vesicles that reloads the empty release sites at the base of the synaptic ribbon (Jackman et al., 2009; Snellman et al., 2011; Chen et al., 2013). The ribbon capacitor will be re-charged with synaptic vesicles in the light phase when vesicle exocytosis is low. This recharged ribbon capacitor will provide release-ready vesicles in the dark when the photoreceptor depolarizes (Jackman et al., 2009; Chen et al., 2013). According to this hypothesis, the rate of release is determined by the rate of vesicle replenishment at the synaptic ribbon. In agreement with this assumption, it was recently shown that major endocytic proteins were strongly enriched in the periactive zone around the synaptic ribbon (Wahl et al., 2013).

### SYNAPTIC CODING AT RIBBON SYNAPSES IN COMPARISON TO CODING IN CONVENTIONAL SYNAPSES

Synaptic coding in ribbon synapses differs from coding in conventional synapses. Conventional synapses are typically phasic synapses that initiate synaptic vesicle exocytosis only when a stimulus, usually an action potential, reaches the presynaptic terminal. The depolarization of the presynaptic terminal opens VGCC, typically P-/Q-/N-type calcium channels in phasic synapses (for review, see Catterall et al., 2005; Striessnig et al., 2010; Catterall, 2011; Südhof, 2012a,b, 2013). The subsequent Ca^2+^-influx initiates synaptic vesicle fusion at the active zone (for review, see Südhof, 2012a,b, 2013). In contrast, ribbon synapses are tonically active synapses that continuously fuse synaptic vesicles with the presynaptic plasma membrane. The basal rate of synaptic vesicle exocytosis is modulated in response to graded changes of the membrane potential. In photoreceptors, exocytosis is high in the dark when the photoreceptor is depolarized. The rate of exocytosis is diminished in the light when the photoreceptor hyperpolarizes (Jackman et al., 2009). Synaptic vesicle exocytosis at ribbon synapses is also initiated by influx of Ca^2+^ through VGCC. But ribbon synapses typically use other Ca^2+^-channels than phasic synapses (see below). As mentioned above, exocytosis of synaptic vesicles preferentially occurs at the base of the synaptic ribbon (Zenisek et al., 2000; Chen et al., 2013). Ribbon synapses can fuse several hundreds of synaptic vesicles per second and ribbon (Heidelberger et al., 2005; Schmitz, 2009). Rod photoreceptors maintain continuous exocytosis at physiological membrane potentials indicating a high vesicle release probability in ribbon synapses (Jackman et al., 2009; Chen et al., 2013). How this is achieved is not clear but could be based on a high Ca^2+^-sensitivity of the release machinery and/or an efficient coupling of the release machinery to the presynaptic L-type Ca^2+^-channels (see below). In contrast, release probability in conventional, phasic synapses is typically much lower than in ribbon synapses (for review, see Nicoll and Schmitz, 2005; Rusakov, 2006; Fioravante and Regehr, 2011; Regehr, 2012).

At the single vesicle level, using TIRF microscopy it was shown that synaptic vesicles did not fuse immediately after they appeared at the plasma membrane but paused at the base of the ribbon for about 60 ms before fusion finally occurred (Zenisek et al., 2000; Chen et al., 2013). Sixty-millisecond is a relatively long time delay and indicates that the speed of single vesicle exocytosis cannot be the main determinant for synaptic coding and its temporal fidelity at ribbon synapses.

The retrieval of synaptic vesicles has been recently appreciated as an important aspect of signaling at ribbon synapses (see also above). Continuous exocytosis in photoreceptor ribbon synapses caused depletion of synaptic vesicles at the base of the synaptic ribbon at physiological membrane potentials (Jackman et al., 2009; Chen et al., 2013). This depletion of presynaptic release sites during tonic exocytosis at physiological membrane potentials suggested that synaptic vesicle replenishment is rate-limiting for exocytosis and an important aspect for signaling at ribbon synapses (Jackman et al., 2009; Chen et al., 2013). The rate of vesicle replenishment would thus determine how smooth exocytosis could proceed. Gaps in continuous synaptic vesicle exocytosis at the ribbon synapse, e.g., as a result of insufficient vesicle replenishment, could contribute important signaling informations.

Despite the large physiological differences between ribbon synapses and conventional synapses, these synapses do not differ strongly in the composition of the exocytotic machinery (for review, see Schmitz, 2009; Matthews and Fuchs, 2010; Mercer and Thoreson, 2011; Schmitz et al., 2012). In photoreceptor ribbon synapses, syntaxin 1 is replaced by syntaxin 3b (Curtis et al., 2008, 2010); complexin 1/2 by complexin 3/4 (Reim et al., 2005, 2009); Munc13-1 by ubMunc13-2 (Schmitz et al., 2001; Cooper et al., 2012); piccolo by piccolino (Regus-Leidig et al., 2013). But the key players of exocytosis are remarkably conserved.

It should be mentioned that ribbon synapses are not a uniform population of synapses but display both structural and functional differences (Heidelberger et al., 2005; Moser et al., 2006; Matthews and Fuchs, 2010; Mercer and Thoreson, 2011). Exocytosis in ribbon synapses of hair cells in the inner ear operates in a fundamentally different manner and appears to function without neuronal SNARE proteins (Nouvian et al., 2011).

### DEVELOPMENT OF THE SYNAPTIC RIBBON COMPLEX

Photoreceptor ribbon synapses of the mouse retina develop postnatally (Olney, 1968; Blanks et al., 1974). The assembly of the synaptic ribbon complex in photoreceptors occurs via distinct steps. Starting around postnatal day 4 (P4) small, spherical ribbon precursors are anchored at the arciform density of the photoreceptor active zone as judged by electron microscopy and immunofluorescence microscopy with antibodies against RIBEYE (Olney, 1968; Hermes et al., 1992; Regus-Leidig et al., 2010a,b; Liu et al., 2013; Zabouri and Haverkamp, 2013). Starting around P10 and proceeding with the time of eye opening, the immature, short spherical ribbons develop into the mature form of the synaptic ribbon which is bar-shaped in cross sections and plate-shaped in three-dimensional representations (**Figures 3C,F**; Hermes et al., 1992; Vollrath and Spiwoks-Becker, 1996; Adly et al., 1999; Schmitz et al., 2006; Regus-Leidig et al., 2010a,b; Liu et al., 2013).

Recent studies demonstrated that VGCC have a key role for the developmental organization of the presynaptic cytoskeleton including the synaptic ribbons.

### SYNAPTIC RIBBONS AND VOLTAGE-GATED Ca^2+^ CHANNELS

L-type VGCC are clustered at the basal end of the synaptic ribbon (Heidelberger et al., 2005; Doering et al., 2007; Striessnig et al., 2010; Mercer and Thoreson, 2011; Mercer et al., 2011a; Schmitz et al., 2012). The site is a hotspot of exocytosis (Zenisek et al., 2000). Entry of Ca^2+^ through L-type VGCC is essential for triggering exocytosis at the photoreceptor synapse (Dacheux and Miller, 1976; Schmitz and Witkovsky, 1997; Witkovsky et al., 1997; Heidelberger et al., 2005). The VGCC at the active zone of photoreceptors consists of a pore-forming transmembrane α1 subunit (Cav1.4, in photoreceptors; Cav1.3 in inner hair cells), an auxiliary β2-subunit and the α2δ4-subunit (Ball et al., 2002, 2011; Catterall et al., 2005; Morgans et al., 2005; Wycisk et al., 2006; Buraei and Yang, 2010; Striessnig et al., 2010; Catterall, 2011; Mercer et al., 2011a; Schmitz et al., 2012; Liu et al., 2013). Few Cav-channels at the base of the synaptic ribbons are sufficient to drive exocytosis (Brandt et al., 2005; Bartoletti et al., 2011). L-type VGCC in photoreceptors are well suited to support tonical exocytosis at ribbon synapses because these channels do not show Ca^2+^-dependent inactivation (CDI) and little voltage-dependent inactivation (VDI; Singh et al., 2006; Striessnig et al., 2010; Catterall, 2011; Shaltiel et al., 2012). Furthermore, the Ca^2+^-sensitivity of release is high and exocytosis is triggered by low, submicromolar Ca^2+^-concentrations (Rieke and Schwartz, 1996; Thoreson et al., 2004; Heidelberger et al., 2005; Sheng et al., 2007; Choi et al., 2008; Jarsky et al., 2010). In addition to L-type channels, retinal bipolar cells also express transient, T-type VGCC (Pan et al., 2001; Ma and Pan, 2003; Hu et al., 2009).

In the development of the outer retina, Cav-channels associate early with synaptic ribbon precursors and synaptic ribbons (Liu et al., 2013; Zabouri and Haverkamp, 2013). Synaptic ribbons do not form properly, if the L-type calcium channels are missing, e.g., in Cav1.4 mouse knockout models (Mansergh et al., 2005; Chang et al., 2006; Raven et al., 2007; Lodha et al., 2010). In the different available Cav1.4 mouse knockout models, the Cav1.4-channel was shown to exert a strong, severe influence on synaptic ribbon localization and assembly. Some quantitative differences were observed in the different available Cav1.4 mouse knockout models (Mansergh et al., 2005; Chang et al., 2006; Raven et al., 2007; Lodha et al., 2010).

Two recent studies (Liu et al., 2013; Zabouri and Haverkamp, 2013) analyzed in detail the relevance of the Cav-channels for distinct steps of synaptic ribbon assembly at the active zone and for the subsequent maturation of the synaptic ribbon complex (Liu et al., 2013; Zabouri and Haverkamp, 2013). Initially, at early stages of development, immature ribbon precursors are clustered in the terminal and probably anchored to the active zone (Zabouri and Haverkamp, 2013) even if the Cav-channels are missing. But – in contrast to the wildtype – these immature ribbon precursors are not aligned and thus functionally linked with postsynaptic glutamate receptors in the Cav1.4 knockout (Liu et al., 2013). Furthermore, without proper channel activity, these immature ribbon precursors do not mature into the bar-shaped synaptic ribbons but remain immature (Liu et al., 2013; Zabouri and Haverkamp, 2013). Interestingly, normal physiological activity of the Cav-channel is needed to transform immature ribbons into mature ribbons. Both loss-of-function mutants as well as gain-of-function mutants (Knoflach et al., 2013; Liu et al., 2013) of the Cav-channels prevented proper maturation of the synaptic ribbon complex (Liu et al., 2013).

Similarly, also knockout models of various proteins linked to the Cav1.4-channel (i.e., β2-subunit of Cav1.4 channels, CaBP4, bassoon, and laminin β2 (for review, see Nishimune, 2012) displayed absent, smaller or non-attached “floating” synaptic ribbons (Libby et al., 1999; Ball et al., 2002; Dick et al., 2003; Haeseleer et al., 2004; Nishimune et al., 2004; Nishimune, 2012). In agreement with these findings, voltage-gated Cav-channels in the inner ear were shown to regulate synaptic ribbon size (Frank et al., 2009, 2010; Sheets et al., 2011, 2012).

Zabouri and Haverkamp (2013) demonstrated that Cav-channels are not only important for the proper development of the synaptic ribbon complex but also for the development of the presynaptic cytoskeleton in photoreceptors. Deletion of Cav1.4 lead to misassembly of many components of the presynaptic photoreceptor cytoskeleton including PSD95, the plasma membrane Ca^2+^-ATPase (PMCA), Veli3, β-dystroglycan, bassoon, and RIM2. As consequence, the architecture of the synaptic terminal was profoundly altered.

These data argue that the Cav1.4 channel in photoreceptors is a central organizer of the photoreceptor ribbon synapse active zone in general and for the synaptic ribbon complex in particular. Several molecules are candidates that could provide a link between synaptic ribbons and voltage-gated Cav1.4 calcium channels. The RIM multi-domain family of proteins are essential components of the active zones in conventional and ribbon synapses (Wang et al., 1997; Südhof, 2013). In conventional active zones, they are found at presynaptic densities; in ribbon synapses both at the active zone (arciform densities; RIM2; tom Dieck et al., 2005) and also at the proper synaptic ribbon (RIM1, tom Dieck et al., 2005). RIMs are important for various central aspects of synapse function (Deng et al., 2011; Han et al., 2011; Kaeser et al., 2011; for review, see Südhof, 2013). RIM proteins are crucial for anchoring P/Q/N-type VGCC at the active zone in conventional synapses via a direct PDZ-domain interaction (Deng et al., 2011; Han et al., 2011; Kaeser et al., 2011; for review, see Südhof, 2013). RIMs indirectly interact with L-type Ca^2+^-channels via RIM-BPs (Hibino et al., 2002). Besides immobilizing VGCC, RIMs are essential for vesicle priming via interaction with Munc13 and for synaptic vesicle docking via interaction with rab3/rab27 (Deng et al., 2011; Han et al., 2011; Kaeser et al., 2011; for review, see Südhof, 2013).

RIM1 has also been reported to be associated with the β-subunits (β4, β2a) of VGCC (Kiyonaka et al., 2007; Miki et al., 2007). This interaction was suggested to suppress inactivation of VGCC (Kiyonaka et al., 2007; Miki et al., 2007). Interestingly, a point mutation in the C2A-domain of RIM1 is responsible for a cone rod-dystrophy (CORD7; Johnson et al., 2003). CAST/ELKS proteins that are also present at the synaptic ribbon complex have been recently reported to be also associated with β-subunits of VGCC (Chen et al., 2011; Kiyonaka et al., 2012). Similarly, bassoon has been described to be associated with β-subunits of VGCC (Chen et al., 2011; Nishimune et al., 2012). The binding of bassoon to β-subunits of VGCC is compatible with the finding that the organization of VGCC was disturbed in inner ear ribbon synapses of bassoon-deficient mice (Frank et al., 2010). Thus, the active zone with the synaptic ribbons appears to be intimately associated with VGCC and organized by these channels by a multitude of different protein–protein interactions.

### PRESYNAPTIC Ca^2+^, GCAP2, AND SYNAPTIC RIBBON DYNAMICS

In the mature adult mouse retina, synaptic ribbons also underlie changes in size. These changes are illumination-dependent and appear to be regulated by Ca^2+^ (Vollrath and Spiwoks-Becker, 1996; Adly et al., 1999; Spiwoks-Becker et al., 2004; Schmitz, 2009; Regus-Leidig et al., 2010a). Several studies observed a light-induced reduction in the size of synaptic ribbons in the mouse retina (Adly et al., 1999; Spiwoks-Becker et al., 2004; Regus-Leidig et al., 2010a; Fuchs et al., 2013). The bar-shaped ribbons appear to disassemble (and re-assemble) from the free cytosolic, non-membrane-anchored end (**Figure 3**). From this end of the bar-shaped ribbon, spherical ribbons, the synaptic spheres, detach (**Figure 3**). Thus, this illumination-dependent growth and disassembly of the synaptic ribbon appears to employ similar intermediate structures as in development (**Figure 3C**). These spherical intermediates are similar in appearance as the spherical synaptic ribbons of hair cells in the inner ear (for review, see Moser et al., 2006). The synaptic spheres are still associated with numerous synaptic vesicles (**Figures 3A–C**). Spherical ribbons were added to remaining ribbons during darkness (Spiwoks-Becker et al., 2004; Regus-Leidig et al., 2010a).

The light-induced reduction of synaptic ribbon size could be mimicked by lowering intracellular Ca^2+^ (Adly et al., 1999; Spiwoks-Becker et al., 2004) suggesting that Ca^2+^ is an important factor that regulates ribbon size. In fact, while lowering intracellular Ca^2+^ lead to a reduction of synaptic ribbon size, increases of intracellular Ca^2+^ resulted in increased ribbon sizes (Spiwoks-Becker et al., 2004; Regus-Leidig et al., 2010a). The genetic background of animals also influences synaptic ribbon dynamics. Synaptic ribbon size plasticity has been reported to be stronger in albinotic mice than in pigmented mice (Fuchs et al., 2013).

Mechanisms of assembly and disassembly of synaptic ribbons in the mature retina are still largely unknown. The NCS protein GCAP2 is involved in the Ca^2+^-dependent disassembly of synaptic ribbons (Venkatesan et al., 2010; López-del Hoyo et al., 2012). RIBEYE, the main component of synaptic ribbons, binds the NCS protein GCAP2 (Venkatesan et al., 2010). GCAP2 is the only known Ca^2+^-binding protein of synaptic ribbons. Interaction between RIBEYE and GCAP2 occurs via the B-domain of RIBEYE and the C-terminal region (CTR) of GCAP2 (Venkatesan et al., 2010; for review, see Schmitz et al., 2012). The CTR of GCAP2 is divergent in sequence between GCAP1 and GCAP2. In agreement, GCAP1 does not bind to RIBEYE while GCAP2 does (Venkatesan et al., 2010). Recent ultrastructural analyses, using postembedding immunogold electron microscopy demonstrated that GCAP2 is a component of synaptic ribbons that is directly localized at the synaptic ribbon body (López-del Hoyo et al., 2012). Viral overexpression of GCAP2 in organotypical retina cultures leads to a disassembly of synaptic ribbons (Venkatesan et al., 2010). Similarly, in GCAP2 – overexpressing transgenic mice, synaptic ribbons also tend to disassemble more easily than in the wildtype retinas (López-del Hoyo et al., 2012) although the synaptic ribbon complex as such is normally formed in GCAP2 knockout mice.

Low intracellular Ca^2+^-concentrations lead to dimerization and also to conformational changes in the CTR of GCAP2 (Olshevskaya et al., 1999; Peshenko et al., 2004). In particular, low intracellular Ca^2+^ leads to exposure of the CTR of GCAP2 (Peshenko et al., 2004). In the Ca^2+^-free form of GCAP2, the CTR is exposed while in the Ca^2+^-bound state it is not (or much less; Peshenko et al., 2004). Therefore, RIBEYE–GCAP2 interaction is most likely favored by conditions of low intracellular Ca^2+^, e.g., at illumination. Furthermore, while RIBEYE–GCAP2 interaction is promoted by NAD(H) (Venkatesan et al., 2010), NADH destabilizes the RIBEYE–RIBEYE interaction network (i.e., RE(A)-RE(B) interaction ; Magupalli et al., 2008) which would lead to an overall tendency of reduced RIBEYE–RIBEYE interaction. By this mechanism, depletion of Ca^2+^ from GCAP2 could contribute to the disassembly of synaptic ribbons in photoreceptor synapses. By this way of thinking, GCAP2 binding to the synaptic ribbon could thus be involved in the regulation of the Ca^2+^-dependent dynamics of synaptic ribbons.

**FIGURE 4:**
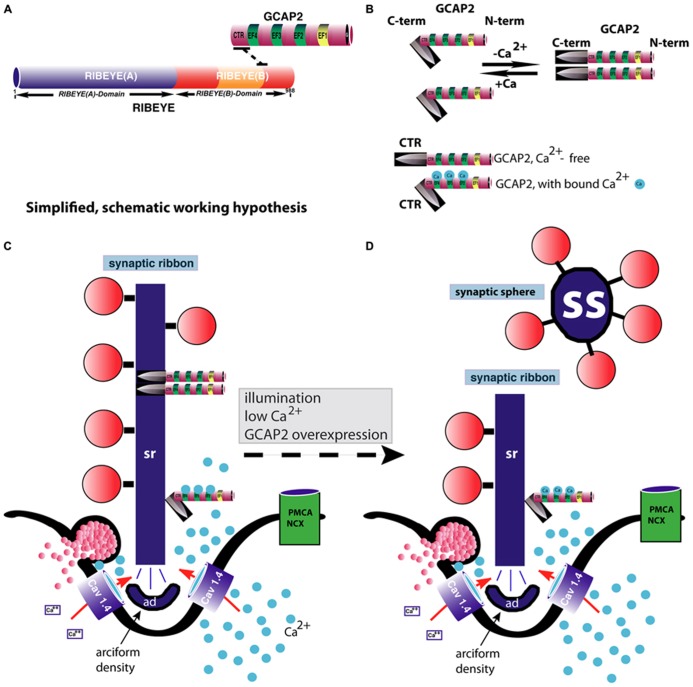
**GCAP2-mediated disassembly of the synaptic ribbon.** **(A)** Previous studies have shown that GCAP2 interacts with RIBEYE, the main component of synaptic ribbons (Venkatesan et al., 2010). The drawing in **(A)** schematically summarizes the interaction between GCAP2 and RIBEYE. The C-terminal region (CTR) of GCAP2 interacts with the hinge region of RIBEYE(B)-domain that connects the NAD(H)-binding sub-domain with the substrate-binding sub-domain of RIBEYE (Venkatesan et al., 2010; Schmitz et al., 2012). This interaction is promoted by NAD(H). **(B)** GCAP2 performs a Ca^2+^-dependent conformational change of its C-terminal region (CTR). In the Ca^2+^-free form, the CTR is exposed, whereas in its Ca^2+^-bound form it is less exposed (Peshenko et al., 2004). In addition, GCAP2 dimerizes at low Ca^2+^ (Olshevskaya et al., 1999). The CTR is responsible for the binding to RIBEYE (Venkatesan et al., 2010). **(C,D)** Binding of GCAP2 to RIBEYE of synaptic ribbons might lead to severing of the synaptic ribbon via exposure of the CTR. Exposure of the CTR occurs if Ca^2+^ is low, e.g., at illumination or if intracellular Ca^2+^ is buffered away by chelators. As a consequence, ribbons are getting shorter. Spherical ribbons, synaptic spheres, dissociate from the free cytosolic end of the synaptic ribbon. While NAD(H) favors binding of GCAP2 to RIBEYE, it weakens RIBEYE–RIBEYE interactions which might ease disassembly of the synaptic ribbon (Magupalli et al., 2008). For sake of clarity, intracellular membrane system, e.g., ER, and mitochondria which could influence intracellular Ca^2+^-levels were omitted in the schematic drawing. These compartments can be expected to further shape the Ca^2+^-signal in the presynaptic terminal. PMCA and NCX-proteins are schematically depicted. The C-terminus (CTR) of GCAP2 that interacts with RIBEYE and which is exposed particularly in the Ca^2+^-free form of GCAP2 is schematically depicted as a knife-like structure because it is proposed to severe the synaptic ribbon structure. The spheres colored in blue in **(B–D)** represent Ca^2+^-ions. sr, synaptic ribbon; ss, synaptic sphere; CTR, C-terminal region of GCAP2; PMCA, plasma membrane Ca^2+^-ATPases; NCX, Na^+^/Ca^2+^-exchanger; ad, arciform density (i.e., the presynaptic density of photoreceptor ribbon synapses); EF, EF-hand.

The conformational change of GCAP2 induced by low Ca^2+^ could thus lead to a severing and subsequent disassembly of the ribbon body into spherical structures, the synaptic spheres (**Figure 4**). The immunogold electron microscopic studies by López-del Hoyo et al. (2012) showed that GCAP2 is not homogeneously distributed along the entire ribbon body (as is RIBEYE; Schmitz et al., 2000), but is clustered at hotspots at the synaptic ribbon surface. These hot spots of GCAP2 immunoreactivity on the synaptic ribbon could represent sites of ribbon disassembly (**Figure 4**).

### INTRACELLULAR MODULATION OF Ca^2+^-SIGNALS IN PHOTORECEPTOR TERMINALS

Ca^2+^ signals generated by influx through VGCC are modulated by various intracellular mechanisms that can subsequently increase or decrease cytosolic Ca^2+^-concentration. The final Ca^2+^-signal in the presynaptic terminal is shaped by the temporal and spatial interactions of these mechanisms (Krizaj and Copenhagen, 2002; Szikra and Krizaj, 2006).

The smooth endoplasmic reticulum (ER) is a main intracellular Ca^2+^-source (Parekh, 2011) and located close to the synaptic ribbon in photoreceptor ribbon synapses as judged by immunolabeling with antibodies against ER components, e.g., the sarco(endo)plasmic reticulum Ca^2+^-ATPase (SERCA)-2 protein (Johnson et al., 2007; Babai et al., 2010). Ca^2+^-induced Ca^2+^-release (CICR) from the ER can amplify Ca^2+^-signals and enhance signal transmission (Krizaj et al., 1999; Krizaj, 2003; Cadetti et al., 2006; Suryanarayanan and Slaughter, 2006; Szikra and Krizaj, 2007; Szikra et al., 2008, 2009; Babai et al., 2010). Several studies showed that CICR contributes to tonic release at the photoreceptor synapse (Krizaj et al., 1999; Cadetti et al., 2006; Suryanarayanan and Slaughter, 2006; Babai et al., 2010). Both classes of ER-localized receptors responsible for mobilization of Ca^2+^ from the ER, i.e., ryanodine receptors (RyR) and IP3 receptors (IP3R) have been localized to photoreceptor terminals (Peng et al., 1991; Krizaj et al., 1999; Krizaj, 2003). CICR can also activate Ca^2+^-activated Cl^-^-channels at the presynaptic plasma membrane. TMEM16B-type of Ca^2+^-activated Cl^-^-channels have been localized to the photoreceptor terminal (Lalonde et al., 2009; Stöhr et al., 2009; Mercer et al., 2011b). Activation of Ca^2+^-activated Cl^-^-channels in photoreceptor terminals should lead to depolarization because the membrane potential of about ≈–40 mV at darkness is more negative than the Cl^-^-equilibrium potential of -20 mV in rod photoreceptor synapses (Thoreson et al., 2002; Babai et al., 2010).

If intracellular Ca^2+^-stores are exhausted, store-operated calcium entry (SOCE) mechanisms can replenish them (for review, see Parekh and Putney, 2005; Lewis, 2011; Soboloff et al., 2012; Gudlur et al., 2013). The ER-localized EF-hand-containing Ca^2+^-sensor protein STIM activates Ca^2+^-selective, plasma membrane located Ca^2+^-release-activated Ca^2+^ (CRAC)-channels in case of store-depletion. CRAC channels contain Orai-proteins as major components that allow Ca^2+^ influx through the plasma membrane and subsequent Ca^2+^ replenishment of the emptied ER store (Lewis, 2011; Soboloff et al., 2012; Gudlur et al., 2013). TRPC1 proteins also contribute to CRAC channel activity (Szikra et al., 2009; Molnar et al., 2012). Silencing of TRPC1 by RNA interference abolished SOCE in rods but not in cones indicating that TRPC1 could be an important component of SOCE in rods (Szikra et al., 2008). STIM1 proteins have been recently localized to the photoreceptor terminal (Szikra et al., 2009). The SERCA2 ATPase (see below) has been suggested to be the “third element” of SOCE that mediates influx of Ca^2+^ into the ER after it has entered the cytosol via the CRAC channels (Manjarrés et al., 2010, 2011; Alonso et al., 2012). Store-operated Ca^2+^-channels could contribute to sustained Ca^2+^ signals and extend the dynamic range of signaling in the tonically active photoreceptor ribbon synapse.

The PMCAs represent a family of transmembrane proteins (Carafoli, 1999; Tempel and Shilling, 2007) that extrudes Ca^2+^ from the cytosol into the extracellular space in an ATP-dependent manner (Carafoli, 1999; Tempel and Shilling, 2007). The PMCA is immobilized in the plasma membrane of the presynaptic terminal that is scaffolded by PSD95, MPP4, and VELI3 (Aartsen et al., 2006, 2009; Yang et al., 2007; Förster et al., 2009). Proper localization of PMCA in the presynaptic plasma membrane also depends on VGCC (Xing et al., 2012). The PMCA has a strong influence on presynaptic Ca^2+^-signaling (Duncan et al., 2006; Szikra and Krizaj, 2006) and also on synaptic ribbon length dynamics (Yang et al., 2007; Aartsen et al., 2009). Deletion of the MPP4 gene leads to a loss of PMCA from the plasma membrane of the presynaptic photoreceptor terminals (Yang et al., 2007; Aartsen et al., 2009). The subsequent loss of extrusion activity leads to increased intracellular Ca^2+^ and enlarged synaptic ribbons (Yang et al., 2007; Aartsen et al., 2009). Na^+^/Ca^2+^ exchangers (NCXs) extrude Ca^2+^ from the cytosol into the extracellular space under physiological conditions (Lytton, 2007). NCX proteins are closely localized to the synaptic ribbon and thus can be expected to strongly influence synaptic transmission and ribbon dynamics (Duncan et al., 2006; Johnson et al., 2007). PMCA appears to be predominantly expressed in rod spherules; NCX is predominantly expressed in cone pedicles (Johnson et al., 2007). The SERCA can lower intracellular Ca^2+^ by pumping Ca^2+^ from the cytosol into the ER (Bublitz et al., 2013). In photoreceptor synapses, the SERCA pump has been localized in close proximity to the synaptic ribbon (Yang et al., 2007; Babai et al., 2010). The described Ca^2+^-extruding systems are important to restore resting levels of Ca^2+^ after depolarization-induced increases (Duncan et al., 2006; Szikra and Krizaj, 2006; Johnson et al., 2007; Mercer and Thoreson, 2011; Wan et al., 2012; Bublitz et al., 2013). Photoreceptor terminals often contain also mitochondria in proximity to the synaptic ribbon (Johnson et al., 2007). Mitochondria have been suggested to be able to take up large amounts of Ca^2+^ (Rizzuto et al., 2004; Parekh and Putney, 2005; Rizzuto and Pozzan, 2006). In ribbon terminals, Ca^2+^-uptake by mitochondria was found to be only functionally relevant if the Ca^2+^-load was particularly high (Zenisek and Matthews, 2000).

### THE SYNAPTIC RIBBON SIZE IN PHOTORECEPTOR SYNAPSES: A PROXY FOR PRESYNAPTIC Ca^2+^-CONCENTRATION?

As summarized above, there are multiple links between Cav-channels, presynaptic Ca^2+^-levels, synaptic signaling and the synaptic ribbons. Ca^2+^-flux through VGCC as well as the subsequent cross-talk to other Ca^2+^-regulating systems in the presynaptic terminal affect structural plasticity and developmental maturation of the synaptic ribbon in photoreceptor synapses. In the light, when Ca^2+^ is low in presynaptic photoreceptor terminals (Thoreson et al., 2004; Choi et al., 2005, 2008; Sheng et al., 2007; Jackman et al., 2009), synaptic ribbons appear to be smaller than in the dark (Adly et al., 1999; Balkema et al., 2001; Spiwoks-Becker et al., 2004; Hull et al., 2006; Regus-Leidig et al., 2010a). Similarly, in knockout mouse models in which extrusion of Ca^2+^ is disturbed and presynaptic Ca^2+^ increased, synaptic ribbons were longer than in control animals (Yang et al., 2007; Aartsen et al., 2009). On the other hand, in mouse knockout models where Cav-channels were deleted, synaptic ribbons were smaller and did not develop into mature, large synaptic ribbons. Therefore, the size of the synaptic ribbon could appear as a readout of presynaptic Ca^2+^. A recent publication (Liu et al., 2013) indicated, that the Ca^2+^-mediated dynamics of synaptic ribbons – at least in development – is more complex. Also gain-of-function mutations of the Cav1.4 channel, which lead to an activation of the Cav1.4 channel already at more negative membrane potentials, displayed shorter than normal synaptic ribbons (Liu et al., 2013). Future investigation need to provide a more detailed picture about the assembly and disassembly of the synaptic ribbon, its regulation and its consequences for signaling at the photoreceptor synapse.

## SUMMARY AND OUTLOOK

Biochemical and molecular analyses identified the protein composition of synaptic ribbons to a large extent. Surprisingly, most of the ribbon proteins are not only present in ribbon synapses but also in conventional synapses. RIBEYE is the only major building block unique to synaptic ribbons. Still, many questions concerning the structure, assembly, and function of synaptic ribbons remain to be elucidated. How is the assembly of synaptic ribbons regulated? How do immature ribbons develop into mature ribbons? How does Ca^2+^ exerts its influence on synaptic ribbons? Does Ca^2+^-dependent phosphorylation of ribbon proteins play a role? Why are there so many links between presynaptic Ca^2+^-channels and synaptic ribbons? How can very few ribbon synapse-specific components build such a fundamentally different synapse? How are the synaptic ribbon components mechanistically linked to the proposed functions of synaptic ribbons? The application of innovative physiological tools and the analysis of mouse knockout models are needed to answer these questions.

## Conflict of Interest Statement

The author declares that the research was conducted in the absence of any commercial or financial relationships that could be construed as a potential conflict of interest.

## Abbreviations

CNG: cyclic nucleotide-gated channel
CRAC: Ca^2+^-release-activated Ca^2+^-channels
GCAP2: guanylate cyclase-activating protein 2
IS: inner segments
NAD(H): designates both the oxidized (NAD^+^) as well as the reduced form (NADH) of nicotinamide adenine dinucleotide
OS: outer segments
PMCA: plasma membrane Ca^2+^-ATPases
NCX: Na^+^/Ca^2+^-exchanger
RIM: rab3-interacting molecule
RIM-BP: RIM-binding protein
ret-GC: retinal guanylate cyclase
SERCA: sarco/endoplasmic reticulum Ca^2+^-ATPase
SOCE: store-operated Ca^2+^-entry
TIRF: total internal reflection fluorescence
VGCC: voltage-gated Ca^2+^-channels
RE(A): RIBEYE(A)-domain
RE(B): RIBEYE(B)-domain
